# Next-Generation Biomedical Microwave Antennas: Metamaterial Design and Advanced Printing Manufacturing Techniques

**DOI:** 10.3390/s26020440

**Published:** 2026-01-09

**Authors:** Maria Koutsoupidou, Irene S. Karanasiou

**Affiliations:** Division of Mathematics and Engineering Sciences, Department of Military Sciences, Hellenic Army Academy, 16673 Athens, Greece

**Keywords:** biomedical antennas, biomedical imaging, biomedical sensing, metamaterials, additive manufacturing, antenna development, 3D printing, 2D conductive printing

## Abstract

**Highlights:**

**What are the main findings?**

- Additive manufacturing and printed conductive processes (inkjet, aerosol jet, and screen printing) now enable conformal, biocompatible antennas on textiles, elastomers, and biodegradable substrates for wearable and implantable biomedical systems.
- Metamaterial and metasurface techniques significantly improve miniaturization, electromagnetic coupling, gain, and tissue isolation, supporting optimized operation under realistic body-loading conditions.

**What are the implication of the main findings?**

- These technologies support biomedical antennas that are more compact, adaptive, and mechanically compliant, allowing seamless integration with the human body.
- The resulting devices enable higher performance in continuous monitoring, diagnostics, wireless power transfer, and therapeutic applications, accelerating the development of next-generation healthcare systems.

**Abstract:**

Biomedical antennas are essential components in modern healthcare systems, supporting wireless communication, physiological monitoring, diagnostic imaging, and therapeutic energy delivery. Their performance is strongly influenced by proximity to the human body, creating challenges such as impedance detuning, signal absorption, and size constraints that motivate new materials and fabrication approaches. This work reviews recent advances enabling next-generation wearable and implantable antennas, with emphasis on printed electronics, additive manufacturing, flexible hybrid integration, and metamaterial design. Methods discussed include 3D printing and inkjet, aerosol jet, and screen printing for fabricating conductive traces on textiles, elastomers, and biodegradable substrates, as well as multilayer Flexible Hybrid Electronics that co-integrate sensing, power management, and RF components into thin, body-conforming assemblies. Key results highlight how metamaterial and metasurface concepts provide artificial control over dispersion, radiation, and near-field interactions, enabling antenna miniaturization, enhanced gain and focusing, and improved isolation from lossy biological tissue. These approaches reduce SAR, stabilize impedance under deformation, and support more efficient communication and energy transfer. The review concludes that the convergence of novel materials, engineered electromagnetic structures, and AI-assisted optimization is enabling biomedical antennas that are compact, stretchable, personalized, and highly adaptive, supporting future developments in unobtrusive monitoring, wireless implants, point-of-care diagnostics, and continuous clinical interfacing.

## 1. Introduction

RF, microwave, and millimeter-wave antennas are essential components in modern healthcare, supporting applications ranging from physiological monitoring and medical imaging to wireless communication and therapeutic interventions. Biomedical antennas can be broadly classified according to their primary function. Antennas for sensing and therapy, such as those used in microwave imaging, radiometry, hyperthermia, or physiological parameter monitoring, require close coupling with biological tissues to ensure efficient power transfer. In contrast, antennas for communication and power transfer, including wearable and implantable devices, are generally designed to minimize the adverse effects of tissue-induced reactive near-field loading while maintaining safe operation with low Specific Absorption Rate (SAR) values. Consequently, the design of biomedical antennas is shaped by the unique electromagnetic, mechanical, and biocompatibility requirements imposed by the human body, including operating frequency, directivity, size, and material selection.

To address these challenges, researchers have increasingly explored metamaterial and metasurface techniques, which introduce subwavelength features, composite packaging materials, and engineered matching media to enhance antenna performance, enable miniaturization, and tailor the electromagnetic response to specific tissues. In parallel, additive manufacturing (AM) techniques—including fused deposition modeling (FDM), stereolithography (SLA), selective laser sintering (SLS), and charge-programmed deposition (CPD)—have transformed antenna fabrication by enabling rapid prototyping, patient-specific customization, and the realization of complex three-dimensional structures. AM facilitates mechanically flexible, compact, and biocompatible antennas while integrating conductive and dielectric components into a single monolithic structure, supporting graded substrates for impedance tuning and reducing material waste and assembly complexity. Inkjet printing further enables high-resolution deposition of conductive inks on flexible and biocompatible substrates, making it particularly suitable for wearable and minimally invasive biomedical devices.

The convergence of metamaterial concepts, 3D printing and 2D printing techniques provides a versatile framework for developing next-generation biomedical antennas that satisfy stringent requirements for size, flexibility, biocompatibility, and electromagnetic performance. Over time, biomedical antennas have evolved from rigid, planar microstrip designs on conventional PCB materials to flexible, body-conforming structures on polymeric and textile substrates. Early antennas based on ceramic or FR4 materials were limited by mechanical rigidity and suboptimal biocompatibility, prompting a shift toward flexible platforms such as polyimide, polydimethylsiloxane (PDMS), and wearable textiles [1,2]. These substrates enable conformal integration with the human body and maintain performance under mechanical deformation. For instance, stretchable antennas fabricated using silver nanoparticle inks on PDMS exhibit robust operation under strain for wearable 5G applications, while implantable conformal antennas have leveraged metallic orthopedic implants [3], such as titanium hip prostheses, as integrated radiating structures for real-time telemetry at 2.4 GHz [4].

These advances highlight the limitations of traditional subtractive manufacturing and underscore the transformative potential of additive and printing-based approaches, including screen printing, inkjet printing, Aerosol Jet Printing (AJP) and 3D fabrication. Such techniques enable direct patterning of conductive traces onto complex or soft substrates with improved scalability, reduced material waste, and enhanced integration with other biomedical components. The combined innovation of materials and fabrication technologies continues to drive the development of wearable, implantable, and minimally invasive biomedical systems.

Several reviews have addressed biomedical antennas, metamaterial-based antennas, and wearable [5] or implantable RF devices [6] from either an electromagnetic design or an application-oriented perspective. However, these topics are often treated in isolation. In contrast, this review adopts a co-design perspective that integrates (i) electromagnetic functionality through metamaterials and metasurfaces; (ii) fabrication and material realization via advanced printed and additive manufacturing technologies (including 3D printing, inkjet, aerosol jet, and screen printing); and (iii) system-level biomedical considerations such as tissue interaction, biocompatibility, flexibility, and integration with sensors and electronics. This unified perspective highlights how electromagnetic design, fabrication, and biomedical constraints jointly shape next-generation biomedical antennas. Furthermore, the review incorporates emerging directions such as Flexible Hybrid Electronics (FHE), biodegradable and transient RF devices, bioprinting-enabled platforms, and AI-assisted optimization, which are rarely covered together in existing surveys.

The remainder of the paper is organized as follows. Section 2 reviews the main classes of biomedical antennas and their application-dependent requirements. Section 3 surveys metamaterial and metasurface techniques for antenna miniaturization, coupling control, and tissue isolation. Section 4 discusses advanced fabrication technologies, including 3D additive manufacturing, 2D printed and FHE platforms and their role in multilayer and implantable antenna systems. Section 5 highlights emerging trends and open research challenges, and Section 6 concludes the paper.

## 2. Biomedical Antennas Characteristics

**Figure 1:**
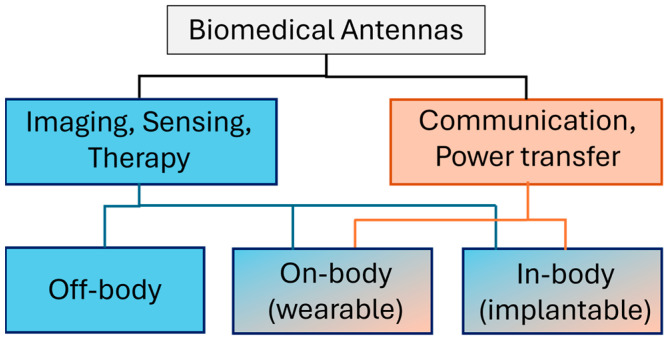
Types of biomedical antennas categorized by application and placement relative to the body.

Biomedical antennas can be broadly categorized according to their intended function: antennas for sensing and imaging, and antennas for wearable or implantable communication. Each category has distinct design considerations dictated by the interaction between the antenna and biological tissue. Figure 1 graphically presents the biomedical antennas classification based on application and placement relative to the body.

### 2.1. Antennas for Sensing and Imaging

Antennas designed for biomedical sensing and imaging applications are often closely coupled to or embedded within biological tissues, where efficient electromagnetic energy transfer is critical. In these cases, the antenna must be carefully matched to the target tissue to maximize signal penetration and sensitivity. Tissue-matched antennas are employed for a variety of physiological monitoring applications, including glucose [7,8,9], lactate [10], and water content measurement [11], as well as temperature and electrical conductivity sensing [12,13]. Additionally, arrays of antennas are utilized for imaging and detection purposes, such as monitoring strokes and aneurysms or detecting tumors in various tissues, such as the breast [14], brain [15], and abdomen [16]. In these scenarios, the near-field interaction with the tissue is leveraged to enhance coupling, improve imaging resolution, and ensure that the electromagnetic field is efficiently absorbed or reflected for diagnostic and therapeutic purposes.

### 2.2. Biomedical Antennas for Communication and Power Transfer

In contrast, antennas intended for communication and power transfer applications, while on-body—mainly on wearable and implantable devices—are typically designed not to match the tissue load [17]. Their primary goal is to establish reliable telemetry, data transfer or interconnection between devices within or on the body, rather than energy absorption by tissues. Examples include antennas for medical telemetry systems that link multiple sensors or devices across the body [18], as well as antennas for wireless power transfer to implants or ingestible devices [19,20,21]. In these cases, minimizing undesired interactions with the high-loss tissue environment is essential to maintain signal integrity, efficiency, and user safety.

### 2.3. Design Challenges and Considerations

Regardless of the specific application, all biomedical antennas face common challenges arising from the highly lossy and heterogeneous nature of biological tissues, which strongly influence near-field properties, input impedance, and radiation patterns. For antennas used in sensing and imaging, this tissue interaction is often exploited to maximize sensitivity, whereas for communication antennas, it must be carefully mitigated to preserve efficiency and reliable signal transmission [22]. In other words, with the exception of implantable antennas, while sensing and imaging antennas are designed to couple strongly with tissues to enhance measurement or treatment efficacy, communication and power-transfer antennas prioritize minimizing tissue interaction to maintain efficient data or energy delivery. Adding further complexity, human tissues exhibit substantial inter- and intra-individual variability in dielectric properties, such as permittivity and conductivity [23]. These variations can shift resonant frequencies, reduce gain, and alter impedance matching, posing particular challenges for implantable and wearable antennas that require consistent performance across diverse users and conditions. Interestingly, the presence of the tissue load can also broaden the antenna’s resonant bandwidth, and lower its quality factor (Q), making it more resilient to minor detuning or frequency shifts.

In addition to electromagnetic considerations, biomedical antennas must meet stringent requirements for size, material selection, biocompatibility, and mechanical flexibility, particularly in wearable and implantable systems. Advances in AM, flexible biocompatible substrates, and metamaterial-inspired designs have facilitated antennas that are both physically compact and mechanically conformal to the body, while maintaining targeted electromagnetic performance [24]. Mechanical conformity to the tissue, besides the comfort, ensures good matching with the tissues, enhancing the coupling without the requirement of thick matching mediums [25]. Consequently, the central challenge in biomedical antenna design lies in balancing tissue interaction, miniaturization, mechanical compliance, and reliable electromagnetic behavior to address a wide spectrum of applications—from diagnostic imaging and sensing to wireless communication and therapeutic interventions.

Overall, the most critical parameter in designing and testing biomedical antennas is the tissue itself, as it governs several key effects: (i) resonance shifts in resonant antennas, (ii) broader bandwidth due to Q-factor reduction, (iii) reduced radiation efficiency, (iv) altered radiation patterns with strong reflection if the antenna is not matched to the tissue, (v) opportunities for antenna miniaturization directly linked to resonance shifts, and (vi) the necessity for biocompatible materials and high-permittivity dielectrics in substrates, packaging and matching medium [26,27,28]. Consequently, the design methodology for biomedical antennas must account for the host or adjacent tissue, including its dielectric properties, shape, volume, and size, to ensure optimal performance in the intended operational environment. Table 1 summarizes the most high-level antenna design characteristics to be considered depending on the specific application and intended location of the antenna.

## 3. Metamaterial Antennas for Biomedical Applications

Metamaterial and metasurface techniques employ artificially engineered subwavelength structures—either metallic, dielectric, or hybrid—embedded within or printed onto a host substrate to modify and enhance the electromagnetic behavior of antennas. These structures enable the realization of properties not typically attainable with conventional materials, allowing antennas to exhibit exotic characteristics or superior performance.

In antenna engineering, metamaterial concepts are commonly applied for several key purposes: (i) to achieve miniaturization by effectively reducing the antenna’s electrical size; (ii) to enhance radiation control by manipulating directivity and beam focusing; and (iii) to improve impedance matching to a target load or, conversely, to enhance isolation from nearby objects or tissues in the near field. For biomedical applications, these last two functionalities are of particular importance and depend on the operational context. Antennas designed for sensing or imaging biological tissues benefit from strong electromagnetic coupling and good impedance matching with the tissue, which ensures efficient power transfer and sensitivity. In contrast, wearable or on-body antennas often require enhanced isolation from the body to minimize detuning and signal absorption, thereby improving communication, power transfer efficiency and user safety.

Beyond these general advantages, the development of metamaterial-based biomedical antennas often aims to tailor the electromagnetic response of conventional radiating elements to the distinct dielectric and dispersive properties of human tissues. This adaptability supports the design of antennas optimized for specific individuals or localized anatomical regions. Additionally, metamaterials can be engineered to introduce advanced functionalities, such as multi-frequency operation, polarization diversity, or even non-linear and non-predictable polarization states, thereby extending the performance range of biomedical systems.

In Table 2, basic metamaterial and metamaterial-inspired structures used for biomedical antennas are classified, and their core functions, advantages, and limitations are summarized. For clarity and reference, recent publications employing these types of metamaterials are provided. Additionally, two recent review papers discuss biomedical metamaterial antennas [22] and antennas for implantable devices [6]. In the following paragraphs, the three primary functions of metamaterials in biomedical antennas—for sensing, imaging, and wearable telecommunication applications—are described.

### 3.1. Miniaturization

**Figure 2:**
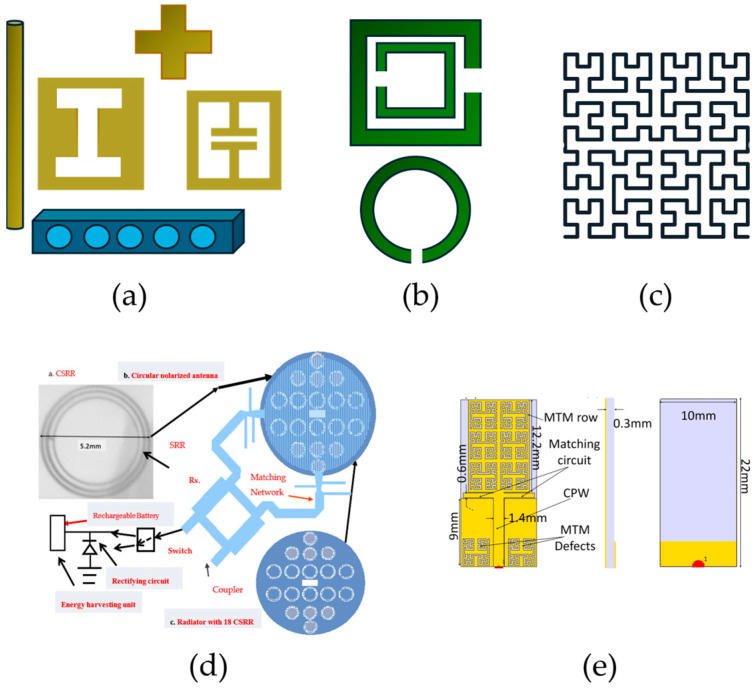
Examples of unit cells of metamaterials used in antenna design: (**a**) Artificial dielectric materials and electric LC (ELC) metamaterials, consisting of dielectric or metallic subwavelength structures—such as rods, strips, crosses, or H-shaped slots—embedded in or on dielectric substrates. (**b**) SRRs are mainly used in artificial magnetic materials. (**c**) A 2D square filled with a Hilbert fractal curve; fractal structures are considered metamaterial-inspired, as they involve subwavelength elements used for miniaturization or the addition of resonances. (**d**) A circularly polarized sensor with an energy harvester incorporating a radiator of an array of circular SRRs (CSRRs), increasing gain by 2–3 dB and bandwidth [31]. (**e**) The front, side and back view of a low-profile, flexible wearable antenna designed for multiple ISM bands for remote health-monitoring applications, using a patch with complementary Minkowski fractal geometry to achieve multiband behavior and miniaturization [34].

Metamaterial techniques have proven highly effective in achieving antenna miniaturization for biomedical applications, where compactness and conformity to the human body are crucial. These approaches exploit arrays of subwavelength resonant elements—such as split-ring resonators (SRRs), complementary Minkowski or Hilbert fractal geometries, and interdigital or E-shaped resonators—to manipulate the effective electromagnetic properties of the antenna’s substrate and surrounding medium. By introducing artificial inductive and capacitive loading, these structures enable the creation of strong localized resonances, allowing the antenna to operate efficiently at lower frequencies without increasing its physical size. For example, SRR-based designs for the radiating elements have been used to achieve λ/10-size antennas for cancer cell sensing, enhancing sensitivity while maintaining compactness, while the integration of Hilbert fractal curves on the ground plane further contributes to size reduction by increasing current path length and enabling multi-resonant behavior [35]. Similarly, complementary Minkowski fractal geometries have shifted the first resonance from 1.25 GHz to 0.4 GHz in wearable communication antennas, demonstrating significant miniaturization [34] (Figure 2e). A triple SRRs array on the substrate was used to bring the cut-off frequency of the two-slotted dipole antenna from 1.8 GHz to 1.2 GHz and also to improve the overall performance of the microwave head imaging system [33]. In implantable and wearable systems, metamaterial-inspired elements such as E-shaped or concentric ring resonators have been incorporated into substrates or superstrates to reduce the antenna footprint while preserving impedance matching and radiation efficiency [30]. Collectively, these metamaterial techniques enable compact, efficient, and frequency-agile biomedical antennas, ensuring seamless integration into small wearable devices and miniature implants.

### 3.2. Coupling and Gain Enhancement

**Figure 3:**
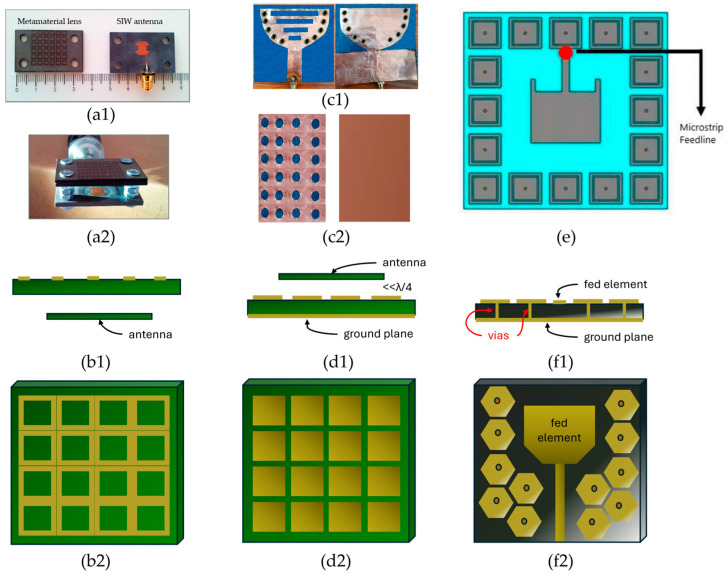
Metamaterial and metamaterial-inspired structures for wearable and biomedical antennas: (**a1**) A metamaterial lens and a Substrate Integrated Waveguide (SIW) antenna for tissue matching at 10.6 GHz and (**a2**) the integrated setup [45]. (**b1**) Schematic placement relative to an antenna and (**b2**) setup of an FSS based on a rectangular loop array functioning as a superstrate. (**c1**) A wearable cavity-backed SIW antenna with (**c2**) an AMC reflector plane on cotton fabric, achieving a gain enhancement of 1.4 dB and SAR reductions of 0.672 W/kg on the human spine and 0.341 W/kg on the forelimbs at 2.45 GHz [55]. (**d1**) Schematic placement relative to an antenna and (**d2**) setup of an AMC substrate based on a rectangular patch array, placed below the antenna at a distance significantly less than λ/4 to operate as a PMC. (**e**) A dual-band wearable mushroom-like EBG-based antenna, providing enhanced gain and −3 dB beamwidth at 2.4 GHz and 5.4 GHz [56]. (**f1**) Schematic placement relative to an antenna and (**f2**) setup of an EBG metamaterial substrate based on hexagonal mushroom patches for surface-wave suppression.

Metamaterial and metasurface concepts have also been widely applied to enhance the gain, focusing capability, and radiation efficiency of biomedical antennas, particularly in microwave imaging and power transfer applications. By engineering subwavelength resonant structures—such as concentric ring arrays, H-shaped slot patches, and cross-slotted loops—researchers have achieved precise control over the electromagnetic field distribution and directivity of the antenna [47,48,49] (Figure 2). These artificial structures act as spatial phase shifters or electromagnetic lenses, concentrating energy toward targeted tissue regions or implanted devices. For instance, a Frequency Selective Surface (FSS) consisting of a 6 × 6 array of SRRs, functioning as a 2D metamaterial lens, was used to improve matching with the tissue [34] (Figure 3(a1,a2)). The incorporation of concentric square-ring radiators has yielded up to 6 dBi gain enhancement in biomedical imaging setups, improving both image resolution and penetration depth [50]. Similarly, vertically stacked SRRs loaded with capacitors have demonstrated up to 280% improvement in power transfer efficiency, even under misaligned conditions, by enhancing near-field coupling and magnetic flux confinement [32]. H-shaped slot patches implemented on high-permittivity superstrates have also been shown to improve the 3 dB axial ratio bandwidth by 6%, demonstrating that metamaterial configurations can simultaneously enhance gain and circular polarization characteristics [29]. A ring-array superstrate placed above an implantable receiver antenna demonstrated a $S_{21}$ improvement for wireless power transfer to implants by a resonance shift [44]. Circular loops in low flexible dielectric have been used to improve overall $S_{21}$ with maximum improvement of 5.4 dB [51]. A further example in gain enhancement shows that adding a loop antenna with cross-slots on the superstrate achieved ~4 dBi gain improvement for biomedical use [52]. Collectively, these metamaterial-based focusing techniques enable biomedical antennas to achieve stronger, more directional radiation patterns and improved energy transfer efficiency, which are essential for high-resolution imaging, targeted diagnostics, and reliable communication with implantable or wearable devices.

### 3.3. Tissue Isolation and SAR Reduction

**Figure 4:**
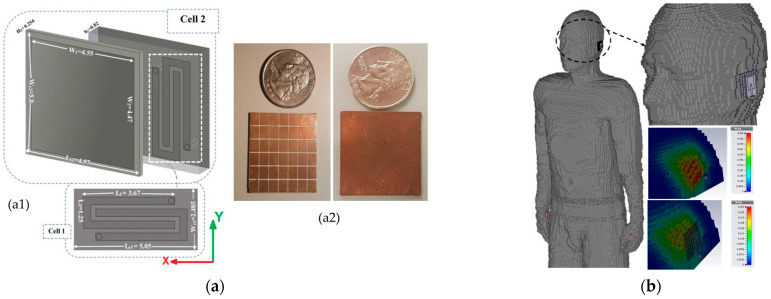
(**a1**) A unit-cell and (**a2**) the front and back viewes of the respective developed MD-AMC substrate for a coplanar waveguide antenna [38]. (**b**) Simulation results for SAR calculation using a head voxel model of the coplanar waveguide antenna without and with the MD-AMC substrate [38].

In wearable and biomedical antenna systems, one of the key challenges is the strong electromagnetic interaction between the antenna and the high-loss biological tissue, which can lead to impedance detuning, reduced radiation efficiency, and elevated SAR levels. To mitigate these effects, metamaterial-inspired FSS (Figure 3(b1,b2)), Artificial Magnetic Conductor (AMC) (Figure 3(c1,c2,d1,d2)) and Electromagnetic Band-Gap (EBG) (Figure 3e(f1,f2)) structures have been widely employed as isolation layers between the antenna radiator and the body. EBGs and AMCs, which can be considered FSSs with a conductive ground plane, act as high-impedance surfaces that reflect incident electromagnetic waves in phase, effectively suppressing surface currents and minimizing backward radiation toward the tissue within a specific frequency band. These configurations not only enhance antenna gain and front-to-back ratio but also, by extension, significantly reduce SAR, and overall improve user safety and communication reliability [37,42,43,53]. Figure 4 presents SAR simulation results for a coplanar waveguide antenna designed for 5G applications [38]. The results demonstrate that a magneto-dielectric AMC (MD-AMC) substrate reduces the maximum SAR averaged over 10 g of tissue from 0.87 W/kg to 0.26 W/kg [38]. Beyond communication applications, such metamaterial structures can also be applied to sensing and imaging biomedical antennas to reduce back radiation and enhance directivity toward the tissue, depending on the antenna’s orientation [54]. FSS- and AMC-backed textile and flexible antennas maintain stable performance under bending and deformation, making them ideal for integration into clothing or skin-mounted sensors [39,40,46]. Furthermore, AMC ground planes can serve as secondary radiators, reinforcing constructive interference and thus improving overall radiation efficiency even in proximity to the body. This dual functionality—enhanced isolation and gain—enables metamaterial-based AMC and FSS structures to serve as an effective solution for wearable antennas, ensuring robust operation, reduced tissue loading, and compliance with safety standards [41].

## 4. Antennas Developed with Advanced Printing Techniques

Lately, AM has been proposed to offer the ability to produce complex and customized antenna structures, reducing lead times and material waste [57]. AM allows for the integration of multiple functionalities into a single antenna structure, such as combining antennas with sensors or other electronic components. However, it is important to select appropriate materials to ensure the desired electromagnetic properties and mechanical performance of the antenna [58]. For instance, the conductivity of the selected materials is essential for the antenna’s functionality, such as impedance and efficiency. Additionally, the selected plastics for the substrates or mold structures can be flexible, biocompatible, durable or degradable, while the dielectric properties are significant during the antenna design.

### 4.1. Common and Novel 3D Printing Techniques for Antenna Fabrication

#### 4.1.1. Fused Deposition Modeling (FDM)

FDM is a widely utilized AM technique that employs thermoplastic materials to construct objects layer by layer. A plastic filament, usually Polylactic Acid (PLA) [59], Acrylonitrile Butadiene Styrene (ABS) [60], Nylon [61], Polyethylene terephthalate glycol (PETG) and Thermoplastic polyurethane (TPU) [62] and the more recent flexible NinjaFlex [63,64], are used to build antenna substrates and molds for 3D complex antenna structures [65,66].

While this method is particularly advantageous in antenna substrate and structure development due to its cost-effectiveness and accessibility, it does not cover the metallic parts of the antenna. For this reason, researchers usually use different metallization methods such as sputter deposition, i.e., a layer of copper over a thin layer of titanium [67], conductive paint [68], galvanic plating [69] or the low-cost option of adhesive metallic tape.

**Figure 5:**
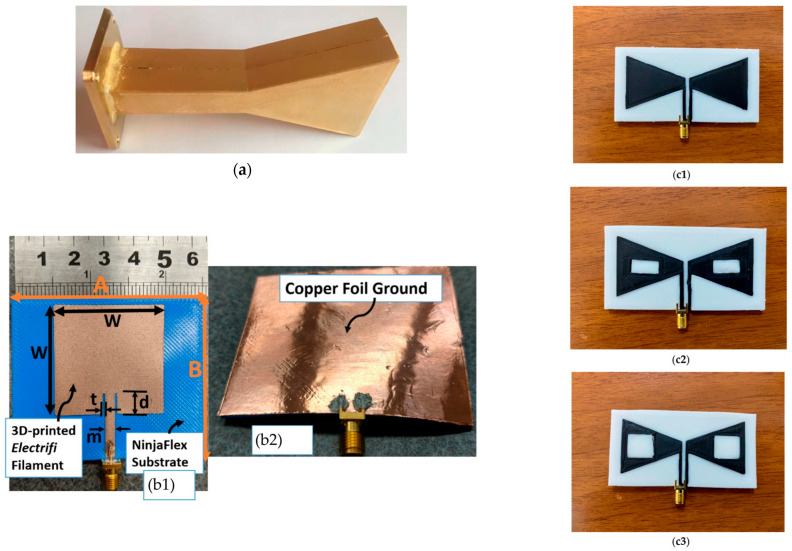
Examples of antennas developed with FDM: (**a**) a horn 3D printed antenna with a gold-plated surface operating at 9.5 GHz, that is significantly lighter than a standardized metallic antenna [69]. (**b1**) A microstrip conformal patch antenna at 2.4 GHz: the prototype was made with NinjaFlex for the substrate and the Electrifi conductive filament for the match, while (**b2**) copper foil was used for the ground plane [64]. (**c1**) A solid and (**c2**,**c3**) two slotted bow-tie antennas were developed using PLA 3D-printed substrates and a graphene-based filament (BlackMagic) for the conductive parts, achieving ultra-wideband (UWB) operation from 2 to 10 GHz, while the conductivity of the graphene could be dynamically tuned by adjusting its chemical potential [71].

Alternatively, researchers have explored the incorporation of conductive fillers into the base polymers (Figure 5). Metallic-type FDM filaments have been investigated for K-band horn antennas [70]. However, the most popular conductive filament used for the metallic parts of 3D printed antennas is the Electrifi [64], with other filaments such as Graphene PLA filaments [71] having also been investigated for the antenna conductive part. Their findings indicated that while these materials are cost-effective, the surface roughness and lower conductivity compared to traditional metals can impact the electromagnetic performance of the antennas [72].

#### 4.1.2. Stereolithography (SLA)

SLA is a 3D printing method that cures liquid resin, layer by layer, using a UV source, which has become very attractive for antenna development, because of its high resolution, good surface finish, and capacity for complex designs and structures with intricate internal features. The photopolymerization process enables the creation of cavities, channels, and non-standard geometries that would be extremely difficult to fabricate using traditional methods [73,74].

Another significant advantage of SLA is its high precision and surface finish, especially for antennas operating at high frequencies, where small surface irregularities can affect performance. Additionally, SLA can be used to precisely control the density of lattice structures, enabling the engineering of graded dielectric properties within the antenna substrate [75].

Finally, SLA simplifies antenna assembly by enabling the monolithic fabrication of complex components. Unlike conventional machining, which may require multiple segments to be fabricated and later assembled, SLA can produce an entire antenna structure as a single piece. This approach eliminates the need for multiple alignments, mechanical joints, and seams that often introduce unwanted discontinuities or losses [73].

Despite these advantages, several challenges remain in the use of SLA for antenna development, since additional metallic coatings are often required to create the conductive parts, while metallization processes are challenging for microfeatures that are connected to higher frequencies. Additionally, post-processing stages such as cleaning and UV curing may induce further shrinkage or distortion. Factors such as temperature sensitivity and moisture absorption further complicate their use, while the intrinsic material losses of solid resins affect the antenna performance [76].

#### 4.1.3. Selective Laser Sintering (SLS)

SLS is an additive manufacturing technique that uses a high-power laser to selectively fuse powdered materials layer by layer, producing solid three-dimensional structures, which allows the fabrication of antennas with complex geometries, internal cavities, and integrated components. Antennas with enhanced structural stability and lightweight designs have been developed with SLS [77]. Additionally, the technique has been proposed for complex dielectric-loaded and lattice-based structures [78].

SLS enables high design flexibility, rapid prototyping, and the possibility of combining multiple materials within a single build. However, the technique has limitations, including relatively high surface roughness and potential material porosity, which can affect the electrical performance of antennas. Post-processing, such as surface smoothing or metallization, is often necessary to optimize conductivity and reduce electromagnetic losses.

#### 4.1.4. Charge Programmed Deposition (CPD)

CPD is an emerging additive manufacturing technique that combines 3D printing with surface charge patterning to enable precise, selective deposition of conductive materials. This approach allows antennas to be fabricated with highly controlled conductive paths directly within a dielectric or structural substrate, creating lightweight, compact and complex antennas [76,79]. It allows integrating multiple materials in a single print with dielectric and conductive components coexisting and reducing assembly complexity [80].

However, CPD faces challenges, including the precise control of surface charge distribution and the limited conductivity of printed materials. Overall, CPD represents a promising direction for next-generation antennas, particularly for applications demanding lightweight, compact [81], and multifunctional designs, such as biomedical sensing and wearable electronics.

### 4.2. Novel 2D Printing Techniques for Antenna Fabrication

In addition to volumetric three-dimensional additive manufacturing, a class of planar and conformal additive fabrication techniques has emerged for the realization of ultra-thin, flexible, and lightweight antennas. These two-dimensional (2D) printing approaches rely on the direct deposition of functional inks onto planar, flexible, or curved substrates (Kapton) and are particularly well suited for wearable, epidermal, and textile-integrated biomedical antennas [82,83]. By enabling high-resolution patterning on soft and biocompatible materials, these methods support conformal integration with the human body while maintaining mechanical compliance and electromagnetic performance.

The main 2D printing techniques considered in this context include inkjet printing, AJP, and screen printing, each offering distinct advantages in terms of resolution, scalability, substrate compatibility, and mechanical robustness. The following subsections summarize their operating principles, capabilities, and relevance for antenna development.

#### 4.2.1. Inkjet Printing for Antenna Development

Inkjet-printed antennas are fabricated through the direct, maskless deposition of conductive inks onto a substrate in a digitally controlled manner. In this process, droplets of metallic nanoparticle-based inks or conductive polymer composites are selectively ejected from a nozzle and deposited to form the desired antenna geometry. This contactless and additive approach enables precise patterning on soft and thermally sensitive substrates, making inkjet printing particularly attractive for wearable and biomedical antenna applications requiring lightweight and conformal structures.

The main advantages of inkjet-printed antennas include high spatial resolution, low material consumption, low fabrication cost, and compatibility with flexible and biocompatible substrates [34,84]. However, limitations arise from the relatively low conductivity of printed inks compared to bulk metals, the need for multiple printing passes to increase metal thickness and reduce resistive losses and the requirement for post-processing steps such as thermal or photonic sintering to improve electrical performance and durability. As a result, inkjet-printed antennas for biomedical applications are predominantly employed in short-range communication for wearable sensors and on-body devices [34,84,85,86].

#### 4.2.2. AJP for Epidermal and Conformal Antennas

**Figure 6:**
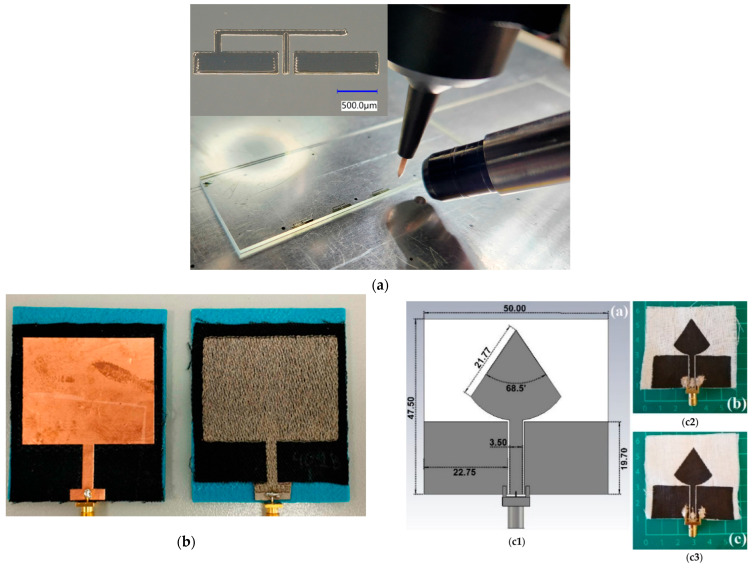
(**a**) Printing multiple 60 GHz PIFAs on a glass substrate using AJP technique using as ink, an aqueous dispersion containing PEDOT:PSS polymer, silver nanowires, high boiling solvents, and surfactants [109]; (**b**) Textile wearable microstrip patch antennas made by copper foil, with conductivity σ = 5.8 × 10^7^ S/m and conductive yarn, with conductivity σ = 1.1 × 10^4^ S/m, that depends on the stitch density [90]; (**c1**) A planar inverted cone textile antenna (CAD model and developed prototypes) for wearable application developed with screen printing using different rations of CNTs/CNC inks: (**c2**) CNTs/CNC—1:1 and (**c3**) CNTs/CNC—1.5:1.5. The operational bandwidth increased from 3.5 to 11.5 GHz (on-body operation) to 2.5–11.5 GHz with increasing CNT concentration [94].

AJP is a high-resolution, non-contact additive process (Figure 6a) capable of depositing ultra-thin conductive traces onto flexible, curved, and soft substrates. This makes it particularly suitable for epidermal antennas realized on biocompatible films (e.g., parylene, silicones) or soft hydrogels. For example, Zhao et al. demonstrated a stretchable dipole antenna formed by injecting liquid metal into a laser-ablated polyacrylamide hydrogel [87]. The high permittivity of the hydrogel reduced the resonant length (approximately 5 cm at 0.93 GHz) and enabled strain-tunable frequency operation in the range of 770–927 MHz [87]. Similarly, Hobbie et al. used a rotating mandrel to conformally print graphene- and CNT-based electronics on an inflated catheter balloon via AJP [88]. These studies demonstrate that AJP enables the fabrication of ultrathin, skin-like wireless circuits on soft substrates and supports multilayer printing on bent or deformable surfaces without the need for complex post-processing. In particular, AJP-printed antennas on hydrogel or polymer films have been suggested as suitable for wearable biomedical telemetry and neuroprosthetic devices.

#### 4.2.3. Screen Printing for Textile and Stretchable Wearable Antennas

Textile wearable antennas are key components of smart garments and on-body sensing platforms, enabling wireless communication while remaining lightweight, flexible, and conformal to the human body. They are used in biomedical applications ranging from health monitoring and fitness tracking to medical telemetry and long-term physiological sensing. Key design challenges include maintaining electromagnetic performance under bending, stretching, and exposure to environmental factors such as sweat and repeated washing [89,90,91]. Various techniques have been developed to integrate antennas into textiles, including embroidery [90,92] (Figure 6b), screen printing and inkjet printing [83], as it has already been described. Embroidery offers precise geometric control using conductive threads, whereas printing-based approaches enable scalable deposition of conductive inks directly onto fabrics. Advanced conductive materials such as carbon nanotubes, silver nanowires, and conductive polymers are commonly employed to achieve high conductivity and mechanical flexibility [92]. Multilayer printing and encapsulation strategies are often used to improve mechanical durability and washability, while electrical interconnections are realized using embroidered vias, eyelets, or snap-on buttons [93].

Screen printing is widely used for the fabrication of textile-based biomedical antennas due to its simplicity, scalability, and compatibility with roll-to-roll processing [94]. In this method, a patterned mesh is used to deposit conductive inks onto fabrics or elastomers. Recent examples include the screen printing of CNT- and polymer-based silver inks onto cotton and polyester substrates. Composite CNT/carboxymethylcellulose inks have been used to realize planar inverted-cone (PICA) and loop antennas on cotton (Figure 6(c1,c2,c3)) [94], while dipole and patch antennas have been demonstrated on cotton–polyester blends using commercial screen-printable inks [95,96].

Such printed antennas can be stretched, bent, or draped over the skin while maintaining acceptable performance. However, washability remains a significant challenge, as resistivity typically increases after repeated laundering. For instance, a five-layer CNT ink printed on cotton exhibited an approximately fivefold increase in resistance after four domestic washing cycles [94] (Figure 6c). Encapsulation using thermoplastic polyurethane (TPU) or polymer coatings is therefore commonly applied to protect printed traces. Despite these challenges, screen printing remains an attractive fabrication route for wearable biomedical antennas owing to its low cost, scalability, and compatibility with textile manufacturing processes.

### 4.3. 3D and 2D Printing Techniques for Biomedical Antennas

The main advantages of additive manufacturing in the domain of electromagnetic biomedical devices are its cost-effectiveness [34], especially for wearable or disposable devices, and its ability to produce complex three-dimensional geometries and custom structures that conform precisely to the contours of human tissue [97]. Moreover, AM enables control over the printed object’s internal density, allowing tuning of the effective dielectric constant. This capability can be exploited to optimize electromagnetic performance and impedance matching of wearable or implantable systems to specific tissue environments [98,99]. These characteristics and their implications for biomedical antenna fabrication are summarized in Table 3. Complementary 2D printing approaches, such as inkjet, aerosol jet, and screen printing, further extend these advantages by enabling the realization of ultra-thin, flexible, and conformal antenna structures directly on soft polymeric or textile substrates, which are particularly relevant for epidermal and wearable biomedical systems. Their main characteristics and possible biomedical antennas application domains are summarized in Table 4.

More generally, AM facilitates rapid prototyping and customization, enabling the development of patient-specific devices such as wearable bands, probes, or helmet-mounted sensors that conform closely to an individual’s anatomy [85]. This personalized approach improves user comfort, reduces motion artifacts, and ensures more consistent sensor performance [97]. The high fabrication speed further allows iterative design optimization without significant time delays or increased production costs.

Given the choice of materials, including biocompatible polymers like PLA, PLGA or PEEK [100], the safe application of wearable and implantable devices is ensured without causing irritation or adverse reactions. In addition, inkjet printing allows the use of polyamide films, which are biocompatible, flexible and mechanically robust, ideal for implantable devices [101]. Similarly, AJP enables deposition on hydrogels and soft elastomers, while screen printing allows scalable integration of antennas into textile substrates, thereby supporting smart garments and long-term wearable health monitoring. Furthermore, AM supports the integration of antennas with other biomedical components, such as sensors [102] or even 3D printed matching media [103], within a single monolithic or hybrid structure. This integration reduces assembly complexity and enhances overall system reliability. Recent work in [104] proposes the application of AM for printing a biosensor, both dielectric and conductive parts, directly inside the human body via a minimally invasive robotic procedure.

Table 5 presents the recent studies on 3D-printed antennas for biomedical sensing, imaging, and communication in wearable systems, summarizing their characteristics, fabrication techniques, employed materials, and the respective design objectives motivating the choice of each manufacturing methodology. The corresponding developments on biomedical antennas based on planar and conformal printing techniques are discussed separately in Table 6.

**Table 3:** Comparison of 3D printing manufacturing processes for biomedical antennas.

3D Printing Technique	Advantages	Disadvantages	Common Substrates	Common Conductive Materials	Accuracy *	Post Processing	Biomedical Antenna Application and Reference
FDM	Low-cost, simple operation, widely available, rapid prototyping	Limited resolution, rough surface finish	Biocompatible polymers, Thermoplastics (PLA, ABS, PETG, TPU, etc.)	Conductive filaments (e.g., graphene/metal composites)	Moderate (100–200 µm)	Surface polishing, conductive path enhancement, thermal annealing	Wearables [99], sensing [103], prototyping [105]
SLA	High resolution, smooth surfaces, complex 3D geometries	High cost, limited material selection, slow process	Photopolymers, biocompatible resins	Metal coating, post-processing	High (25–50 µm)	UV post-curing, cleaning of resin residues, metal coating	Sensing [102],
SLS	Complex 3D structures, no support structures, good mechanical strength	Surface roughness, expensive, limited material choice	Nylon (PA12), biocompatible polymers	Post-deposited metal layers or conductive pastes	Moderate–high (50–150 µm)	Surface smoothing, metallization, thermal stabilization	Complex geometries, liquid antennas **
CPD	Scalable, printing on 3D structures, multi-axis synchronized nozzle system	Lower conductivity, limited thickness, slow process	Ceramic, Flexible substrates	Conductive inks or filaments	Moderate (50–200 µm)	Thermal curing, sintering of conductive tracks	Monolithic development of antennas, Customized wearables [97]

* The reported processing accuracy corresponds to typical values obtained with standard or commercially available equipment. Higher resolutions may be achievable with customized or laboratory-optimized systems. ** To date, no biomedical antennas have been reported using this specific 3D printing technique, although it has been successfully applied to antenna fabrication in non-biomedical applications.

**Table 4:** Comparison of 2D printing manufacturing processes for biomedical antennas.

2D Printing Technique	Advantages	Disadvantages	Common Substrates	Common Conductive Materials	Accuracy *	Post Processing	Biomedical Application
Inkjet Printing	Material-efficient, high resolution	Limited viscosity of inks, slow for large areas	Flexible polymers, textiles	Silver nanoparticle inks, CNT inks	High (10–50 µm)	Thermal or photonic sintering, curing	Wearable antenna, epidermal sensors [85,86]
AJP	High resolution, 3D deposition	Expensive, complex setup	Polymers, glass, flexible substrates	Silver, gold, graphene nanoparticle inks	Very high (5–20 µm)	Thermal or laser sintering, encapsulation	Biomedical devices [88]
Screen Printing on Textiles	Low-cost, scalable	Lower resolution, thickness control limited	Textile substrates	Conductive inks (Ag, Cu)	Moderate (100–200 µm)	Thermal curing, mechanical fixation, encapsulation	Textile wearable antennas [94,96]

* The reported processing accuracy corresponds to typical values obtained with standard or commercially available equipment. Higher resolutions may be achievable with customized or laboratory-optimized systems.

**Table 5:** 3D-printed antennas for biomedical applications.

Ref.	Antenna Type	Technique/3D Printed Part	Material of Dielectric Part	Material of Conductive Part	Operating Band	Biomedical Application	Key Objective
[102]	Slot waveguide antenna	SLA/Antenna structure	FormLabs flexible resin (FLGR02)	Copper sputtered on titanium thin layer	11–13 GHz	Wearable Microfluidics Sensor	Customization and prototyping
[106]	RFID antenna	FDM/ RFID tag	Cotton (fabric)	Graphene Ink, stretchable silver conductor (DuPont PE872)	0.95 GHz	On-body wearable sensors	Prototyping and printing directly on stretchable textiles
[105]	EGRH antenna	FDM/Antenna structure	PLA	Copper plating	0.5–1.3 GHz	Microwave bodyscope	Compact custom design, and dielectric high-K filling for matching
[107]	Planar bow-tie	FDM/substrate	PLA, PLA/copper, PLA/carbon	Copper sheet	Resonances in the 0–3 GHz spectrum	Generic biomedical application	Low-cost and material investigation
[99]	Circularly polarized UHF RFID antenna	FDM/Substrate	PLA	Adhesive copper tape	865–868 MHz	Wearable antenna	To control substrate dielectric permittivity through different printing infill percentages
[108]	Planar monopole	FDM/curved substrate	PLA	Copper tape	3–12 GHz	Generic biomedical application	Intricate 3D structure
[103]	PDRH antenna	FDM/Antenna structure	Polyethylene (PE)	Silver conductive paint	1.5–7 GHz	Abdominal Fat Measurement	Compact custom design, and dielectric high-K filling (TiO_2_) for matching
[97]	Dipole antenna	CDP/FDM for the mold for the conforming substrate	PLA for the mold, ECOFLEX for the substrate	Silver paste	915 MHz and 2.45 GHz	Wearable antenna	Custom design conforming to tissue

**Table 6:** 2D-printed antennas for biomedical applications.

Ref.	Antenna Type	2D Printing Technique	Substrate	Conductive Material	Operating Band	Application	Key Objective
[34]	Coplanar waveguide antenna with meander	Inkjet printing	Flexible polyimide	Silver nanoparticle ink	2.5 GHz	Wearable biomedical sensing	Prototype on flexible substrate
[84]	RFID antenna	Inkjet printing	Textile fabric (cotton/polyester blend)	Silver nanoparticle ink	UHF RFID (~860–960 MHz)	Near-field communication.	Performance and durability of textile RFID antenna under humidity
[86]	Flexible inkjet-printed antenna	Inkjet printing	Flexible polymer film	Conductive Ag nanoparticle ink	2.5 GHz	Wearable electronics	Mechanical robustness and stable RF performance under bending deformation
[85]	Smart bandage with cicruit integrated loop antenna	Inkjet printing	Flexible medical dressing/polymer	Silver nanoparticle ink	2.4 GHz	Wireless wound monitoring	Low-cost antenna integrated into a medical bandage for healthcare monitoring
[87]	Dipole antenna	APJ (femptosecond laser)	Polyacrylamide hydrogel	Liquid metal injected into laser-ablated microchannels	Sub-GHz (~0.9 GHz)	Epidermal/wearable biomedical sensing	Miniaturized, stretch-tunable antenna using hydrogel permittivity and laser-defined microstructures
[95]	Rectangular patch with bandpass filter	Screen printing	Textile substrate	Silver nanoparticle ink	2.5–4.6 GHz	Wearable wireless communication	Integrated filtering and radiation in a single inkjet-printed textile antenna
[94]	Planar inverted cone antenna/loop antennas	Screen printing	Textile fabric	Carbon nanotube ink	3.5–11.5 GHz/2.4 GHz	Smart wearable sensing	Development of textile antennas with mechanical flexibility and washability evaluation
[96]	Coplanar keyhole antenna/RFID tag	Screen printing	Textile fabric	Commercial silver-based ink	5.8 GHz/915 MHz	Wearable wireless communication	Development of textile wearable antennas

The dielectric properties of materials used in 2D and 3D printing are not always well documented over wide frequency ranges and can strongly depend on the specific fabrication process, such as infill ratio in volumetric printing or the number and thickness of deposited conductive layers in printed traces. While several studies have focused on characterizing commonly used materials [94,110,111,112,113], it remains important to rely on experimentally measured material parameters when evaluating performance metrics such as antenna gain and efficiency.

Mechanical and environmental stability are critical considerations for 2D-printed wearable antennas, as their electrical performance can be significantly affected by bending, stretching, moisture, and repeated washing. Wash durability and adhesion of printed conductive patterns have been identified as persistent challenges for textile-based wearable electronics, with dedicated studies highlighting the influence of repeated laundering on conductivity and interfacial stability and the need for enhanced adhesion and protective encapsulation strategies [114]. Cyclic mechanical loading, including bending and flexing over thousands of cycles, has been demonstrated to affect the electrical resistance of printed conductive traces, with high fatigue resistance requiring careful material selection and pattern design [115]. Wearable devices further operate in dynamic environments involving body-related deformation, wrinkling, and fabric care processes such as washing or ironing, which underscores the importance of stability evaluations under realistic usage conditions [94]. In addition, wet or moisture-saturated textiles exhibit significantly altered dielectric permittivity and loss tangent due to water absorption, which can substantially affect antenna impedance, radiation efficiency, and bandwidth [84]. Overall, to mitigate these effects, strategies such as multilayer encapsulation, flexible conductive composites, and surface treatments are often employed to improve mechanical robustness, adhesion, and long-term performance [116,117,118].

### 4.4. Flexible Hybrid Electronics for Implantable and Multi-Layer Antenna Systems

Beyond the fabrication of individual antennas using three-dimensional and two-dimensional printing techniques, increasing attention is being directed toward the system-level integration of antennas with sensors, electronics, and interconnects on flexible platforms. In this context, FHE represents a natural extension of printed and additively manufactured antennas, enabling the co-integration of conventional integrated circuits, sensors, and discrete components with printed interconnects and antennas on flexible, biocompatible substrates [119,120]. This enables compact, multifunctional biomedical modules—such as implants or semi-implants—that can conform to tissue. One representative example is a four-layer flexible “Vertically Separated Multilayer Stretchable Circuit” (VSMSC), in which each layer carries metal traces and components; the bottom layer incorporates sensing pads (e.g., ECG electrodes), while the top layer mounts integrated circuits. This system enabled subcutaneous ECG acquisition, heart-rate monitoring, and on-demand electrical stimulation in a live rat model [121]. Another approach demonstrated the attachment of ultrathin chips and electrodes onto a soft, shape-memory polymer film, comparing surface-mount bonding, embedded ultrathin chips, and through-hole vias to connect neural-stimulation and recording circuits on a flexible substrate [122]. These FHE platforms combine thin-film wiring (often polyimide/Au) with encapsulated silicon dies, sensors, and antennas in stacked configurations, supporting miniaturized implants and hybrid wearable devices. FHEs have been demonstrated for neural interfaces and subcutaneous bioelectronic systems involving sensing and stimulation, with potential for broader biomedical implant integration [121,122].

## 5. Future Trends and Open Research Opportunities

Biomedical antennas are advancing rapidly through developments in printable materials, transient electronics, and AI-driven design. One emerging direction involves the use of 3D-printed metamaterials for sub-millimeter-wave biomedical imaging. A recent study demonstrated a dielectric metalens fabricated by 3D printing that operates in the 0.2–0.9 THz band, offering achromatic and wide-angle focusing for high-resolution terahertz imaging—an innovation that may significantly enhance the compactness and capability of future THz-based diagnostic systems [123].

Another major trend is the rise in biodegradable or transient RF electronics designed for short-term biomedical implants. Verified demonstrations include antennas and wireless power receivers using bioresorbable metals such as magnesium, molybdenum, or tungsten on biodegradable substrates like PLGA. These devices maintain RF performance during the functional lifetime of the implant and safely dissolve afterward, reducing the need for surgical removal and supporting environmentally responsible device design [124].

Multi-material bioprinting is also beginning to influence RF device manufacturing. Early work shows the co-printing of conductive hydrogels or polymers with dielectric scaffolds to produce flexible, body-conforming substrates that can embed antenna traces or passive components. While complete antenna systems produced entirely through this method are still under development, the enabling fabrication techniques have already been validated and point toward future minimally invasive or implantable RF sensors.

At the system design level, machine learning and AI methods are being used to optimize wearable antennas under realistic body-loading conditions. Surrogate-based models and neural network–assisted co-design have been shown to reduce development time and yield antennas with improved bandwidth, impedance stability, and robustness to anatomical variability—long-standing challenges for body-worn RF systems [125].

Finally, multimodal integration of microwave sensing and physiological monitoring is moving toward practical deployment. Millimeter-wave Doppler radar systems have successfully tracked respiratory and cardiac rhythms in real time, providing a contactless alternative or complement to traditional ECG and SpO_2_ monitoring. These platforms combine RF sensing with biomedical signal processing to enable continuous and unobtrusive vital-sign monitoring for wearable or clinical applications [126]. While this review focuses on the RF front-end and electromagnetic design aspects of such systems, downstream physiological signal analysis and feature extraction methods play a complementary role in translating sensed signals into clinically meaningful information.

Collectively, these trends highlight a shift toward printed, adaptive, and intelligent RF systems that interface seamlessly with the human body for next-generation biomedical sensing and diagnostic technologies.

## 6. Conclusions

Advances in materials, manufacturing processes, and electromagnetic engineering are driving a new generation of wearable and implantable biomedical antennas. Additive and printed fabrication techniques, including inkjet, aerosol jet, and screen printing, now enable conformal conductive structures on textiles, elastomers, hydrogels, and biodegradable substrates, supporting scalable, low-cost devices that maintain performance under bending, stretching, and physiological motion. FHEs further allow antennas, sensors, and integrated circuits to be co-fabricated within multilayer platforms, reducing device footprint and improving functional density for minimally invasive and implantable medical systems.

At the electromagnetic level, metamaterial and metasurface concepts provide artificial control over dispersion, impedance matching, and field confinement, enabling antenna miniaturization, improved radiation control, enhanced coupling for sensing, and, conversely, greater isolation from lossy tissue to reduce detuning and specific absorption rate in wearable applications. Their ability to tailor electromagnetic behavior to the dielectric properties of specific tissues also supports personalized device design and advanced functionalities such as multi-band operation and focused beam steering.

By jointly reviewing these developments from the perspectives of electromagnetic design, advanced manufacturing, and biomedical system integration, this work highlights how next-generation biomedical antennas are evolving from isolated RF components into adaptive, personalized, and system-integrated platforms. Combined with emerging machine-learning-based optimization workflows, these technological directions point toward biomedical antennas that are more compact, adaptive, energy-efficient, and seamlessly integrated with the human body, enabling future progress in continuous monitoring, diagnostics, therapeutic delivery, and clinical wireless interfaces.

## Figures and Tables

**Table 1:** General design parameters for biomedical antennas, depending on application and placement relative to the body.

Application	Antenna Placement	Antenna Characteristics and Specifications			
		Size (Miniaturization)	Frequency Bandwidth	Tissue Coupling	Materials
Communication Power Transfer	On-body (wearable)	May be required	Narrow-band and/or multiband: high Q antennas are significantly affected by different tissue properties	Undesired (for linking with external base-free space propagation) Required (for linking with in-body device-tissue propagation)	Biocompatible, including textile
	In-body (implantable)	Required		Required	Biocompatible, coating
Imaging Sensing Therapy	Off-body (near-field/far-field application)	Not required	Depending on the specific application, usually antennas are designed with respect to the targeted tissue	Required	Irrelevant
	On-body (wearable)	May be required		Required	Biocompatible, including textile
	In-body (implantable)	Required		Required	Biocompatible, coating

**Table 2:** Summary of metamaterial categories, structures, and their functional roles in biomedical antennas.

Metamaterial Category	Elements/Structures	Core Function in Antennas	Advantages	Limitations	Selected Recent References
Dielectric Metamaterials and Electric LCs (ELCs)	Rods, crosses, high-permittivity spheres, dielectric resonators, planar LCs	Impedance matching, tissue matching, miniaturization, resonance tuning	Low loss, compact, easy integration in implantable/wearable antennas	Limited bandwidth, ohmic and/or dielectric losses	[29,30]
Magnetic Metamaterials	Split Ring Resonators (SRRs), Omega structures, Loops	Directivity enhancement, miniaturization, artificial magnetism	Strong resonance, compact	Narrow bandwidth, ohmic losses, complex fabrication	[31,32,33]
Fractal/Space-Filling Structures	Hilbert fractal, Minkowski fractal, Koch fractal, etc.	Miniaturization, multi-band operation, impedance matching	Compact, broadband or multi-band, planar integration	Fabrication complexity, sometimes increased ohmic losses at higher frequencies	[34,35,36]
Artificial Magnetic Conductors (AMCs)	Mushroom structures, high-impedance surfaces, planar AMC tiles	Reduce back radiation, surface wave suppression, gain enhancement	Low-profile, directional radiation, improves efficiency	Narrowband, fabrication complexity	[37,38,39,40,41]
Electromagnetic Band-Gaps (EBGs)	Periodic patches, vias, mushroom EBGs, uniplanar compact EBGs	Surface wave suppression, mutual coupling reduction, gain enhancement	Reduces interference, improves radiation efficiency	Complex design, can increase antenna footprint	[42,43]
Frequency Selective Surfaces (FSSs)	Metallic grids, patch arrays, slot arrays, cross-dipole arrays	Shielding, filtering, control of specific frequency propagation, gain enhancement	Wide frequency control can improve directivity	Ohmic losses, limited bandwidth	[44,45,46]

## Data Availability

No new data were created or analyzed in this study.
